# Mistaken Identity: Another Bias in the Use of Relative Genetic Divergence Measures for Detecting Interspecies Introgression

**DOI:** 10.1371/journal.pone.0165032

**Published:** 2016-10-19

**Authors:** Kathryn R. Ritz, Mohamed A. F. Noor

**Affiliations:** Department of Biology, Duke University, Durham, North Carolina, United States of America; Associate Professor, ICELAND

## Abstract

Measures of genetic divergence have long been used to identify evolutionary processes operating within and between species. However, recent reviews have described a bias in the use of relative divergence measures towards incorrectly identifying genomic regions that are seemingly immune to introgression. Here, we present a novel and opposite bias of relative divergence measures: misidentifying regions of introgression between sister species. We examine two distinct haplotypes of intermediate frequency within *Drosophila pseudoobscura* at the *DPSX009* locus. One of these haplotypes had lower relative divergence than another to sister species *D*. *persimilis*. Although we and others initially presumed one haplotype have spread via introgression between *D*. *pseudoobscura* and *D*. *persimilis*, absolute divergence measures and individual sequence analysis suggest that haplotype structuring occurred as the result of within-species processes. The potential for this type of misinference may occur with any haplotype that recently spread within a species. We conclude that absolute measures of genetic divergence are necessary for confirming putative regions of introgression.

## Introduction

One of the most surprising findings since the implementation of molecular evolutionary genetics is that a very large number of species hybridize and successfully exchange genes (See review in [1]). With the increase of high-throughput sequencing availability and more sensitive molecular techniques, a plethora of approaches have been developed to analyze and test for introgression at particular loci. However, investigators often employ the simple and classic approach of examining haplotypes for segregating polymorphisms vs. fixed differences between the species. Several studies specifically report the appearance of introgression based on observed distinct haplotypes, which may appear to be more similar to haplotypes found in the sister taxon [2–4].

Several recent reviews [5–7] have emphasized that the use of relative divergence measures that compare within- to between-species variation, such as F_ST_ or Nei’s D_a_ (average nucleotide divergence between species corrected for average divergence within species [8]), may be misleading with respect to testing for gene flow, particularly in regions of low recombination. Those reviews highlighted several empirical studies that perhaps erroneously attributed variation among regions of the genome in interspecies divergence to interspecies gene flow because they used relative divergence measures. Selective sweeps and background selection reduce variation within species particularly in regions of low recombination, such that the partitioned variation by measures such as F_ST_ and D_a_ exhibits an excess of divergence between species. These low recombination regions then appear to be "islands of divergence” relative to the remainder of the genome, which then incorrectly appears to have experienced gene exchange (the mirage referred to by Noor & Bennett [5]). Absolute divergence measures do not suffer this particular problem, but they can have other confounding issues.

Recently, Gredler *et al*. [9] reported within- and between-species variation at various loci in *Drosophila pseudoobscura* and *D*. *persimilis* across time, and the haplotype structure at one locus (*DPSX009*) showed signals of introgression between these species. At this locus, *D*. *pseudoobscura* harbors two intermediate-frequency haplotypes with several linked differences between them, and one of these haplotypes appears to share more variation with *D*. *persimilis* than with other *D*. *pseudoobscura* haplotypes. Previous work by another group [10] identified this same structuring at *DPSX009*, and noted, "the sequence data suggest the occurrence of gene flow and possibly recent introgression at *X009*.*"* The case for introgression is especially strong because recombination is low around *DPSX009* [11], and thus hitchhiking and background selection around this region should make it unlikely to retain intermediate frequency variation. However, this purported introgression is curious because the mapping studies have shown that *DPSX009* is linked to factor(s) conferring reproductive isolation [12] which should impede introgression, though it is possible factor(s) have evolved more recently than introgression occurred.

Here, we report a more detailed examination of the haplotype structure at this locus. While previous studies have interpreted the pattern of divergence at this locus as introgression [9,10], closer examination and use of absolute divergence measures suggest the haplotype structuring is instead likely a consequence of within-species processes. Our study demonstrates that relative measures of divergence may not only inflate measurements of divergence in areas of low genetic diversity [5,6], but also inflate measurements of introgression in areas that are currently undergoing within-species processes, such as selective sweeps. We do not argue that this particular bias is necessarily common, however we observe it within our case study, and other studies have inappropriately relied heavily upon relative divergence measures in their interpretations [5–7], so we stress that caution is warranted.

## Results and Discussion

### Haplotype structure and comparison to sister species

**Fig 1:**
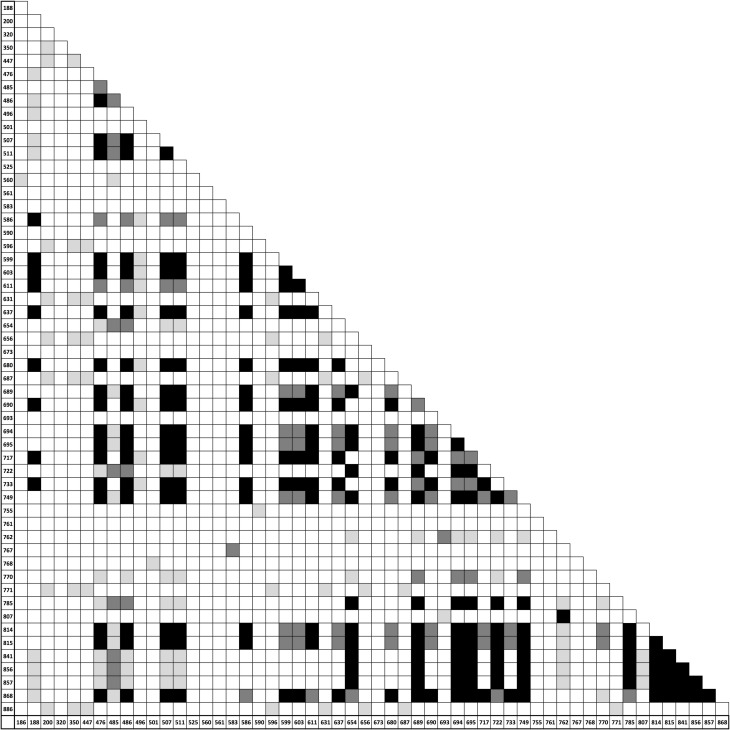
Significant linkage disequilibrium across the *DPSX009* locus in *D*. *pseudoobscura*. Shading corresponds to significant values (light grey: p<0.05, dark grey: p<0.005, and black: p<0.0005). 55 polymorphic sites across 51 sequences were compared using a Fisher’s exact test. Bases range from nucleotide 168 to 886 across the locus (see axes).

*D*. *pseudoobscura DPSX009* sequences from 1997 and 2013 revealed the appearance of a distinct haplotype structure spanning approximately 300 bases [9]. This haplotype contains 7 SNPs and two 15bp indels in complete linkage disequilibrium, an extremely unusual observation in *D*. *pseudoobscura* where LD typically decays to >10% of background level within approximately 20bp [13]. Other SNPs in this region also exhibited strong but incomplete LD extending further out (up to 700bp, see Fig 1). The presence of two haplotype groups at intermediate frequency in the 2013 samples drove the estimate of Tajima’s D to be strongly positive (0.73806; [9]), which contrasts most other loci studied in the species (e.g., [10,14,15]).

Three processes might explain such an observation. First, reduced levels of recombination, perhaps through a microinversion, can cause haplotype structuring within a population. Indeed, previous studies have found that this region of the *D*. *pseudoobscura* genome has unusually low recombination rates relative to the rest of the genome (data in [11,13]). Second, recent introgression from a hybridizing species may result in the observed pattern. *D*. *pseudoobscura* is known to hybridize with its sibling species *D*. *persimilis* in the wild [16] and many studies have documented evidence of gene exchange between them [10,17–19]. The presence of long haplotypes often indicates introgression between species [20,21], and Machado *et al*. [10] report shared partial haplotypes between these two species. Third, within-species processes such a selective sweep in progress or a new balanced polymorphism can cause rapid spread of a previously rare haplotype. This mechanism is potentially consistent with Machado *et al*.'s [10] report of population structuring at this locus, as well as the suggestion of a possible shift in abundance of one of the haplotypes between 1997 and 2013 [9] causing a sign change in Tajima's D to positive. We note that these processes are not mutually exclusive, and that combinations of these evolutionary events may occur simultaneously or sequentially. Here we interpret analyses of the *DPSX009* locus to better understand the pattern observed.

We compared both *D*. *pseudoobscura* haplotype groups to *D*. *persimilis* at the *DPSX009* locus, where the haplotype groups are defined by alleles at two 15-bp indels that are in perfect linkage disequilibrium. One *D*. *pseudoobscura* haplotype (hereafter, haplotype psA) has 1 fixed difference from *D*. *persimilis* and 9 shared polymorphisms, while the other *D*. *pseudoobscura* haplotype (psB) has 10 fixed differences and 2 shared polymorphisms (Fisher's exact test, two-tailed p = 0.019, Table 1), where shared polymorphisms are defined as polymorphic sites with alleles present in both groups [22]. As a consequence of defining haplogroups psA and psB by their genotype at the two 15bp indels, we exclude these indels from further nucleotide diversity analysis, though we acknowledge that excluding them deflates slightly the absolute differentiation between psB and *D*. *persimilis*. Nonetheless, indels are routinely excluded in many population genomic analyses, and coding these indels as SNPs would have created circularity in that we would be comparing divergence between pairs of groups when one group was explicitly defined so as to have greater divergence. We note that the definition of haplotypes from polymorphism data in cases like this is subjective; factors such as window size, number of fixed differences chosen, and recombination may influence classification of samples. We keep this potential for bias in mind throughout our analysis.

**Table 1:** Summary of population genetic measures at the *DPSX009 locus*. Observed haplotype structure within *D*. *pseudoobscura*, divergence measures relative to *D*. *persimilis*, and nucleotide diversity of considered haplotypes.

	Fixed Differences from *D*. *persimilis*		Shared Polymorphisms with *D*. *persimilis*		D_a_ relative to *D*. *persimilis*	D_xy_ relative to *D*. *persimilis*	Nucleotide Diversity (π)
	SNPs	indels	SNPs	indels			
psA	1	0	7	2	0.0100	0.0243	0.0165
psB	7	3	0	2	0.0157	0.0226	0.0029
*D*. *persimilis*	N/A	N/A	N/A	N/A	N/A	N/A	0.0119

Quantifying the nucleotide differences between species relative to variation within species using Nei’s D_a_ [8], we find that D_a_ between psA and *D*. *persimilis* is 0.0100, while D_a_ between psB and *D*. *persimilis* is 0.0157. Such a pattern, where one of two haplogroups found within a species is more similar by relative divergence measures to an abundant haplotype found in another species, could erroneously be interpreted as potential evidence of introgression.

Machado *et al*. [10] noted geographical structuring of variation at *DPSX009* with a small number of samples. We explored this further by genotyping 50 isolates derived from four diverse geographic areas using one of the indels differentiating psA and psB. Both haplotypes psA and psB were present in each population at an intermediate frequency (data in S1 Table), and no significant structuring was apparent (Fisher’s exact test, p = 0.36). Consequently, we test a suite of predictions for whether the observed pattern of divergence results from a recent introgression or within-species processes (Table 2).

**Table 2:** Support for models explaining haplotype structuring observed at *DPSX009*.

Introgression. If the haplotype structuring at this locus is the result of introgression of psA into *D*. *psuedoobscura* from *D*. *persimilis*, we predict the following:		
Hypothesis	Observed	Support for introgression model?
D_xy_ between psA and *D*. *persimilis* is **less than** D_xy_ between psB and *D*. *persimilis*.	D_xy_(psA-*D*. *persimilis*) = 0.0243, D_xy_(psB-*D*. *persimilis*) = 0.0226	NO
π for psA is **less than** π for *D*. *persimilis*.	π(psA) = 0.0165, π(*D*. *persimilis*) = 0.0119	NO
π for psA is **less than** π for psB	π(psA) = 0.0165, π(psB) = 0.00285	NO
D_a_ between *in silico* expanded haplotypes and *D*. *persimilis* is **less than** D_a_ between psA and *D*. *persimilis*.	Mean *in silico*-*D*. *persimilis* D_a_ = 0.0183, D_a_(psA-*D*. *persimilis*) = 0.0100	NO
**Within species processes**. If the haplotype structuring at this locus is the result of within species processes, we predict the following:		
D_xy_ between psA and psB should be 1) **less than** D_xy_ between psA and *D*. *persimilis* and 2) **less than** D_xy_ between psB and *D*. *persimilis*.*	D_xy_(psA-psB) = 0.0217, D_xy_(psA-*D*. *persimilis*) = 0.0243, and D_xy_(pB-*D*. *persimilis*) = 0.0226, Comparison 1: p<0.000001, Comparison 2: p = 0.1204	YES^†^
Fixed differences between psB and *D*. *persimilis* will be derived in psB. They will not be shared with outgroup species *D*. *miranda*.	10 of 10 fixed differences between psB and *D*. *persimilis* are not shared with *D*. *miranda*, and thus are derived (see S1 Fig)	YES

*These patterns may not be strong because of extensive shared variation between *D*. *pseudoobscura* and *D*. *persimilis* given their recent divergence.

^†^Comparison 1 was statistically significant, and comparison 2 was not significant but was in the expected direction. However, the application of a statistical analysis in this instance is subject to a pseudoreplication bias, which we discuss in Materials and Methods.

### Tests for introgression

Previous studies have suggested potential artifacts associated with the use of relative measures of divergence in testing for introgression [5,6]. As such, we examined variation at the *DPSX009* locus using an absolute measure of divergence, Nei’s D_xy_ [8]. If the psA haplotype resembles *D*. *persimilis* due to introgression between these species, we predict D_xy_ should be lower between psA and *D*. *persimilis* than between psB and *D*. *persimilis* (Table 2). In contrast, if one of the two *D*. *pseudoobscura* haplotypes arose and spread as a result of within-species processes, psA and psB should more closely resemble each other than either resembles *D*. *persimilis* (Table 2). We observe that D_xy_ is slightly higher between psA and *D*. *persimilis* than between psB and *D*. *persimilis*, and we find lower sequence difference between psA and psB than between psA and *D*. *persimilis* or between psB and *D*. *persimilis*, thus failing to support the introgression hypothesis prediction and providing some support for the within-species processes hypothesis prediction (summary in Table 2).

Additionally, if the psA haplotype introgressed recently from *D*. *persimilis* into *D*. *pseudoobscura*, we predict that there should be less nucleotide diversity within the newer psA haplotype than the potentially ancestral psB haplotype. The opposite was true (see Table 2), however these predictions potentially assume directionality and a single introgression event rather than multiple introgression events. Further, introgression of psA would likely result in less nucleotide diversity in psA than in a *D*. *persimilis* progenitor haplotype. Again, the opposite was true (see Table 2).

Finally, if the less polymorphic haplotype psB was originally a single haplotype that has spread within *D*. *pseudoobscura* through within-species processes rather than introgression, we predict that most of the differences between psB and *D*. *persimilis* would be derived in psB relative to an outgroup species, whereas the introgression hypothesis does not necessarily make such a prediction. We compared the 10 differences fixed between psB and *D*. *persimilis* to outgroup species *Drosophila miranda*. We found all 10 sites in *D*. *persimilis* shared the *D*. *miranda* allele, consistent with the hypothesis that psB is a newer haplotype whose spread within *D*. *pseudoobscura* resulted from within-species processes (see S1 Fig). The phylogenetic relationship between the *D*. *pseudoobscura*, *D*. *persimilis*, and *D*. *miranda* sequences are depicted in S2 Fig [23].

### Spread of a single haplotype can mimic observed pattern

The above lines of evidence suggest that the presence of two haplotype groups at the *DPSX009* locus in *D*. *pseudoobscura* may be that the haplogroup with less nucleotide diversity (psB) stems from a single, derived *D*. *pseudoobscura* haplotype that recently became more abundant via selection or other within-species processes. Intermediate frequency variation in *D*. *pseudoobscura* is rare, and most molecular variation is present as singleton alleles [10]. Linked singleton differences across a single progenitor psB haplotype may spread in a population and result in a positive Tajima's D overall and a high D_a_ between the new haplogroup and *D*. *persimilis* because of the low nucleotide diversity within psB. To test this hypothesis, we examined how divergence from *D*. *persimilis* is reflected if selected haplotypes within the hypothesized ancestral haplogroup (psA) were to suddenly become abundant (data in S2 Table). We predicted that, if psA is the ancestral haplogroup and prevalence of psB is the result of a within-species spread, individual psA haplotypes spread *in silico* would exhibit a pattern of differentiation similar to that observed for psB: a D_a_ similar to psB and higher than psA relative to *D*. *persimilis*.

Consistent with this prediction, D_a_ from the artificial haplogroup ranged from 0.01262 to 0.0251 (median = 0.01544), with each artificial spread having a D_a_ to *D*. *persimilis* higher than the original psA haplogroup (psA-*D*. *persimilis* D_a_ = 0.0100). The median D_a_ to *D*. *persimilis* across the artificial haplogroups was also highly similar to D_a_ from psB (psB-*D*.*persimilis* D_a_ = 0.0157). We see a similar pattern when indels are coded in the *in silico* analysis as SNPs, where D_a_ ranges from 0.0127 to 0.0256 (median = 0.0163, data in S3 Table).

We can further compare the pattern of divergence observed for psB to the artificial haplogroups by examining the number of fixed differences between each group and *D*. *persimilis*. We see that the *in silico* expansions have between 7 and 17 fixed differences from *D*. *persimilis* (mean = 11.76), a range consistent with that observed for psB (10 fixed differences). Again, this result is consistent with our prediction that the apparent divergence between psB and psA is the result of within-species processes.

We also found that one artificial spread resulted in a highly positive Tajima’s D in the overall population including the expanded haplogroup and the original psA haplogroup (Tajima’s D = 1.5878, data in S2 Table). This observation is similar to the aforementioned pattern seen by Gredler *et al*. (2015) [9]. Our simulations of the expansion of single haplotypes create a pattern of genetic diversity nearly identical to what was observed in natural populations: two intermediate frequency haplotype groups of which one (the ancestral group, psA) appears to be more similar to *D*. *persimilis* by relative divergence measures. From these results, we suggest that the pattern of intermediate haplotype frequency observed at *DPSX009* is likely the result of the spread of a single progenitor haplotype.

## Synopsis

Together, these data indicate that the patterns of variation at *DPSX009* in *D*. *pseudoobscura* are entirely consistent with the action of within-species processes such as selection, and we find an absence of evidence for interspecies introgression. The inference of potential introgression at this locus described by Machado *et al*. [10] and initially hypothesized by us here reflects a bias associated with patterns of intraspecific variation misleading interspecies comparisons, particularly those using relative divergence measures like F_ST_ or D_a_. The exact nature of the within-species processes at work is unclear: rather than a selective sweep in progress, it could represent a new balanced polymorphism or extensive gene flow from an isolated population that experienced a local bottleneck.

The previously identified bias associated with relative divergence measures [5,6] focused on the misleading appearance of high divergence and immunity to introgression in regions of low recombination. Here, we present a distinct and opposite bias: a region of low recombination incorrectly appearing to have introgressed between species. As with the previously reported bias, use of absolute divergence measures can help to determine the strength of evidence for introgression between species at particular loci.

## Materials and Methods

### Sequences and Fly Stocks

*DPSX009* sequences for 37 *D*. *pseudoobscura* and 20 *D*. *persimilis* individuals originating from Mt. Saint Helena, California, USA were downloaded from GenBank (Gredler *et al*. 2015 [9]). Additional *D*. *pseudoobscura* populations were surveyed using flies previously collected by M.A.F. Noor and stored in at -80°C. These populations include 18 isolines from western Washington (Roslyn, Goldendale, and Easton) and 15 isolines from eastern Washington (Cheney), collected in 1996, 8 isolines from American Fork Canyon in American Fork, Utah, collected in 1997, and 9 isolines from Flagstaff, Arizona, collected in 1997. No permits were required for the described study, which complied with all relevant regulations.

### DNA Prep, Amplification, and Genotyping

Genomic DNA from one frozen male individual per isoline was extracted using a single fly squish protocol [24]. Although the lines were slightly inbred, males were chosen to prevent amplification of heterozygous individuals, as *DPSX009* is on the X-chromosome. Primers were designed to span a 15bp insertion within the psA haplotype at *DPSX009*. Individuals were genotyped following PCR and imaging on a 2% agarose in TBE gel.

### Haplotype Classification and Data Analysis

MSH *D*. *pseudoobscura* sequences provided by J. Gredler were aligned using ClustalW in BioEdit 7.0.9 [25] and fixed differences between samples were manually documented and compared across the *DPSX009* locus. A 9-bp microinversion was removed from all sequences to prevent biases in genetic diversity calculations. Samples were then divided into groups by haplotype (psA and psB), and compared to *D*. *persimilis* across the locus. 13 *D*. *pseudoobscura* sequences were classified as psA by the presence of 7 SNPs and two 15-bp indels in complete linkage disequilibrium. The remaining 24 *D*. *pseudoobscura* sequences were classified as psB by the absence of some or all of the 9 diagnostic psA traits.

DNAsp 5.10.01 [26] was used to calculate Nei’s (1987) D_a_, Nei’s D_xy_, and π at *DPSX009* between psA (n = 13) and *D*. *persimilis* (n = 20) and again between psB (n = 24) and *D*. *persimilis* (n = 20). DNAsp calculates Nei’s D_xy_ and Nei’s D_a_ using Nei 1987 equations 10.20 and 10.21, respectively. Fixed differences and shared polymorphisms across compared groups were confirmed manually (data in Table 1). Shared polymorphisms are defined as sites that are polymorphic in both groups. To test for the statistical significance of difference in divergence between each *D*. *pseudoobscura* haplogroup to the other vs. to *D*. *persimilis*, we created a matrix indicating percent sequence difference between every pair of haplotypes. We then bootstrapped (with replacement) the matrix 1,000,000 times and assessed how often average difference between *D*. *persimilis* to either psA or psB was equal to or greater than the difference between psA to psB. The difference between psA-psB vs. psA-*D*. *persimilis* appeared to be highly statistically significant (no resamplings exhibited equal or greater divergence) while that between psA-psB and psB-*D*. *persimilis* was not so (120,416 bootstraps exhibited equal or greater divergence). However, we do not emphasize these outcomes as test statistics because of the issue of pseudoreplication due to what appears to be a recent shared coalescent event within psA. Instead, the weight of evidence from multiple separate comparisons (Table 2) argues for our interpretation of the results.

### Linkage Disequilibrium Across *DPSX009*

DNAsp was used to complete a pairwise Fisher’s exact test between 55 polymorphic sites across 37 MSH *D*. *pseudoobscura DPSX009* sequences (Accession numbers: KM887677.1, KM887676.1, KM887675.1, KM887674.1, KM887673.1, KM887672.1, KM887671.1, KM887670.1, KM887669.1, KM887668.1, KM887667.1, KM887666.1, KM887645.1, KM887644.1, KM887643.1, KM887642.1, KM887641.1, KM887640.1, KM887639.1, KM887638.1, KM887637.1, KM887636.1, KM887635.1, KM887634.1, KM887633.1, KM887632.1, KM887631.1, KM887630.1, KM887629.1, KM887628.1, KM887627.1, KM887626.1, KM887625.1, KM887624.1, KM887623.1, KM887622.1, KM887621.1, AF157576.1). 13 additional *D*. *pseudoobscura DPSX009* sequences were included from NCBI GenBank, published by Machado *et al*. (2002) [10] (Accession numbers: AF450606.1, AF450605.1, AF450604.1, AF450603.1, AF450602.1, AF450601.1, AF450600.1, AF450598.1, AF450599.1, AF450597.1, AF450596.1, AF450595.1, AF450594.1). Pairwise results were piped into Microsoft Excel, where significant comparisons were shaded by value (light grey: p<0.05, dark grey: p<0.005, black: p<0.0005; see Fig 1).

### *In silico* psA Haplotype Expansions

Each of the 13 psA-classified *DPSX009* sequences was expanded *in silico* to simulate an increase in haplotype abundance. These sequences were expanded to the same population size as the available sequences for the psA group (n = 13). Specifically, each sequence with the psA haplotype was copied 13 times to represent a theoretical haplogroup resulting from selection or within-species processes. To prevent exclusion of indels by DNAsp, we repeat analyses with indels coded as SNPs in these sequences. Expanded haplotypes were then compared to *D*. *persimilis* (n = 20) in DNAsp, where D_a_ and D_xy_ were calculated. Fixed differences and shared polymorphisms across each expanded haplotype and *D*. *persimilis* were confirmed manually. Mean D_a_ and D_xy_ values were calculated across the 13 simulations (data in S2 and S3 Tables). We do not discuss the D_xy_ values within our analysis, since this measurement is uninformative here. D_xy_ is always equal to D_a_ plus a constant factor (0.0059, half of π_*D*. *persimilis*_) because there is no variation within the artificial haplogroups.

## Supporting Information

S1 Fig Variable sites across *DPSX009* haplotypes. Summary of fixed differences and shared polymorphisms between psA, psB, and *D*. *persimilis*. Additional alignment to *D*. *miranda* and comparison to fixed differences between psB and *D*. *persimilis* supports hypothesis that psB is derived and results from within-species processes.
(PDF)

S2 Fig Phylogeny of *DPSX009* sequences shows clustering of psA and psB haplotypes. Independent clustering of psB and of psA with *D*. *persimilis* sequences can be seen in this neighbor joining phylogeny generated using phylo.io [23]. *D*. *miranda* is used as an outgroup.
(PDF)

S1 Table Presence of psA and psB haplotype structure in additional populations of *D*. *pseudoobscura*. (DOCX)

S2 Table *In silico* expansion of individual psA haplotypes and comparison to *D*. *persimilis*, with indels excluded. (DOCX)

S3 Table *In silico* expansion of individual psA haplotypes and comparison to *D*. *persimilis*, with indels included. (DOCX)
