# Supplementary material for: Mistaken Identity: Another Bias in the Use of Relative Genetic Divergence Measures for Detecting Interspecies Introgression
Source: PLoS One. 2016 Oct 19;11(10):e0165032. doi: 10.1371/journal.pone.0165032 (PMC5070774; doi:10.1371/journal.pone.0165032)
Supplement: S1 Fig — Summary of fixed differences and shared polymorphisms between psA, psB, and D. persimilis. Additional alignment to D. miranda and comparison to fixed differences between psB and D. persimilis supports hypothesis that psB is derived and results from within-species processes. (PDF) [file pone.0165032.s001.pdf]

	Location	138	447	470	507	511	586	611	631	638	654	657	699	700	701	785	807	815	817	896	
	Description	7bp indel	SNP	SNP	SNP	SNP	SNP	SNP	SNP	1bp indel	SNP	SNP	SNP & 1bp indel	SNP & 1bp indel	15bp indel	SNP	SNP	SNP	15bp indel	1bp indel	
D. persimilis	D. persimilis_2013-78	-	T	G	G	T	C	C	G	-	G	T	G	G	+	C	A	C	-	-	
	D. persimilis_2013-45	+	T	G	C	T	C	C	T	+	A	T	T	G	+	C	A	G	-	-	
	D. persimilis_2013-48	+	T	G	G	T	C	C	T	+	A	T	T	G	+	T	A	G	-	-	
	D. persimilis_2013-24	+	T	G	G	T	C	C	G	-	G	T	G	G	+	C	A	C	-	-	
	D. persimilis_2013-20	+	T	G	G	T	C	C	G	-	A	T	T	G	+	C	A	G	-	-	
	D. persimilis_2013-53	+	T	G	C	T	C	G	G	-	A	T	G	G	+	T	A	G	-	-	
	D. persimilis_2013-26	+	G	G	C	T	C	C	G	-	G	T	G	G	+	T	A	G	-	-	
	D. persimilis_2013-42	+	T	G	C	T	C	G	G	-	G	T	G	G	+	T	A	G	-	-	
	D. persimilis_2013-63	+	T	G	G	T	C	C	G	-	A	T	T	G	+	T	A	G	-	-	
	D. persimilis_2013-17	+	T	G	G	T	C	C	T	+	A	T	T	G	+	T	A	G	-	-	
	D. persimilis_2013-5	+	A	G	G	T	C	G	G	-	A	T	T	G	+	T	A	G	-	-	
	D. persimilis_2013-64	-	T	G	A	T	C	C	G	-	G	T	G	G	+	C	A	G	-	+	
	D. persimilis_2013-33	+	T	G	G	T	C	G	G	-	A	T	G	G	+	T	A	G	-	-	
	D. persimilis_2013-86	-	A	G	G	T	C	C	T	+	A	T	T	G	+	C	A	G	-	-	
	D. persimilis_2013-93	+	T	G	C	T	C	C	G	-	A	T	T	G	+	T	A	G	-	-	
	D. persimilis_1997-42	+	T	G	C	T	C	G	G	-	A	T	T	G	+	C	A	G	-	-	
	D. persimilis_1997-1	+	T	G	G	T	C	C	T	+	A	T	T	G	+	T	A	C	-	-	
	D. persimilis_1997-7	+	T	G	A	T	C	C	G	-	G	T	G	G	+	C	A	G	-	-	
	D. persimilis_1997-3	+	T	G	G	T	C	C	G	-	G	T	G	G	+	T	A	G	-	-	
	D. persimilis_1997-26	+	T	G	G	T	C	C	T	+	A	T	G	G	+	C	A	C	-	-	
***D. pseudoobscura* psA**	D. pseudoobscura_1997-30	+	T	A	G	C	G	C	G	-	G	T	G	G	+	C	G	C	-	-	
	D. pseudoobscura_1997-91	+	A	A	G	C	C	G	T	+	A	C	T	G	+	T	A	C	-	-	
	D. pseudoobscura_1997-4	+	T	A	C	T	C	G	G	+	A	T	T	G	+	T	G	C	-	-	
	D. pseudoobscura_2013-85	-	T	A	G	C	G	C	G	-	G	T	G	A	+	C	G	C	-	-	
	D. pseudoobscura_2013-15	+	T	A	C	T	G	C	G	-	G	T	G	G	+	C	G	C	-	-	
	D. pseudoobscura_2013-79	+	T	A	C	T	G	C	G	-	G	T	G	G	+	C	G	C	-	-	
	D. pseudoobscura_2013-7	+	T	A	C	T	G	C	G	-	G	T	G	G	+	C	G	C	-	-	
	D. pseudoobscura_2013-37	-	T	A	C	T	G	C	G	-	G	T	G	G	+	C	G	C	-	-	
	D. pseudoobscura_2013-1	-	T	A	C	T	G	C	G	-	G	T	G	G	+	C	G	C	-	-	
	D. pseudoobscura_2013-4	+	T	A	C	T	C	G	G	+	A	T	T	G	+	T	G	C	-	-	
	D. pseudoobscura_2013-35	+	T	A	C	T	C	G	G	+	A	T	T	G	+	T	G	C	-	-	
	D. pseudoobscura_2013-60	-	T	A	C	T	C	G	G	+	A	T	T	G	+	T	G	C	-	-	
	D. pseudoobscura_2013-76	+	T	A	C	T	C	G	G	+	A	T	T	G	+	T	G	C	-	-	
***D. pseudoobscura* psB**	D. pseudoobscura_1997-16	+	T	A	G	C	G	C	G	-	A	G	-	-	-	T	G	T	+	-	
	D. pseudoobscura_1997-9	+	T	A	G	C	G	C	G	-	A	C	-	-	-	T	G	T	+	-	
	D. pseudoobscura_1997-13	+	T	A	G	C	G	C	G	-	A	C	-	-	-	T	G	T	+	-	
	D. pseudoobscura_1997-10	+	T	A	G	C	G	C	G	-	A	C	-	-	-	T	G	T	+	-	
	D. pseudoobscura_1997-24	+	T	A	G	C	G	C	G	-	A	C	-	-	-	T	G	T	+	-	
	D. pseudoobscura_1997-31	-	T	A	G	C	G	C	G	-	A	C	-	-	-	T	G	T	+	-	
	D. pseudoobscura_1997-2	+	T	A	G	C	G	C	G	-	A	C	-	-	-	T	G	T	+	-	
	D. pseudoobscura_1997-37	-	T	A	G	C	G	C	G	-	A	C	-	-	-	T	G	T	+	-	
	D. pseudoobscura_1997-32	-	T	A	G	C	G	C	G	-	A	C	-	-	-	T	G	T	+	-	
	D. pseudoobscura_2013-36	+	T	A	G	C	G	C	G	-	A	G	-	-	-	T	G	T	+	-	
	D. pseudoobscura_2013-11	+	T	A	G	C	G	C	G	-	A	G	-	-	-	T	G	T	+	-	
	D. pseudoobscura_2013-65	+	T	A	G	C	G	C	G	-	A	C	-	-	-	T	G	T	+	-	
	D. pseudoobscura_2013-83	+	T	A	G	C	G	C	G	-	A	C	-	-	-	T	G	T	+	-	
	D. pseudoobscura_2013-67	+	T	A	G	C	G	C	G	-	A	C	-	-	-	T	G	T	+	-	
	D. pseudoobscura_2013-3	+	T	A	G	C	G	C	G	-	A	C	-	-	-	T	G	T	+	-	
	D. pseudoobscura_2013-6	+	T	A	G	C	G	C	G	-	A	C	-	-	-	T	G	T	+	-	
	D. pseudoobscura_2013-52	+	T	A	G	C	G	C	G	-	A	C	-	-	-	T	G	T	+	-	
	D. pseudoobscura_2013-84	+	T	A	G	C	G	C	G	-	A	C	-	-	-	T	G	T	+	-	
	D. pseudoobscura_2013-81	+	T	A	G	C	G	C	G	-	A	C	-	-	-	T	G	T	+	+	
	D. pseudoobscura_2013-68	-	T	A	G	C	G	C	G	-	A	C	-	-	-	T	G	T	+	-	
	D. pseudoobscura_2013-29	-	T	A	G	C	G	C	G	-	A	C	-	-	-	T	G	T	+	+	
	D. pseudoobscura_2013-77	-	T	A	G	C	G	C	G	-	A	C	-	-	-	T	G	T	+	-	
	D. pseudoobscura_2013-30	+	T	A	G	C	G	C	G	-	A	C	-	-	-	T	G	T	+	-	
	D. pseudoobscura_2013-18	-	T	A	G	C	G	C	G	-	A	C	-	-	-	T	G	T	+	-	
	D. miranda	-	A	G	G	T	C	C	G	+	A	T	T	G	+	C	A	C	-	-	
	Analysis																				
	**psA to *D. persimilis***																				***Totals***
	Fixed Differences			1																	1
	Shared Polymorphisms	1	1		1			1	1	1	1		1			1					9
	**psB to *D. persimilis***																				
	Fixed Differences			1		1	1					1	1	1	1		1	1	1		10
	Shared Polymorphisms	1																		1	2
	**psB to *D. miranda***																				
	Derived fixed differences between psB and *D. persimilis*			1		1	1					1	1	1	1		1	1	1		10
	% differences derived																				100
