# Supplementary figures and images for: Mistaken Identity: Another Bias in the Use of Relative Genetic Divergence Measures for Detecting Interspecies Introgression
Source: PLoS One. 2016 Oct 19;11(10):e0165032. doi: 10.1371/journal.pone.0165032 (PMC5070774; doi:10.1371/journal.pone.0165032)

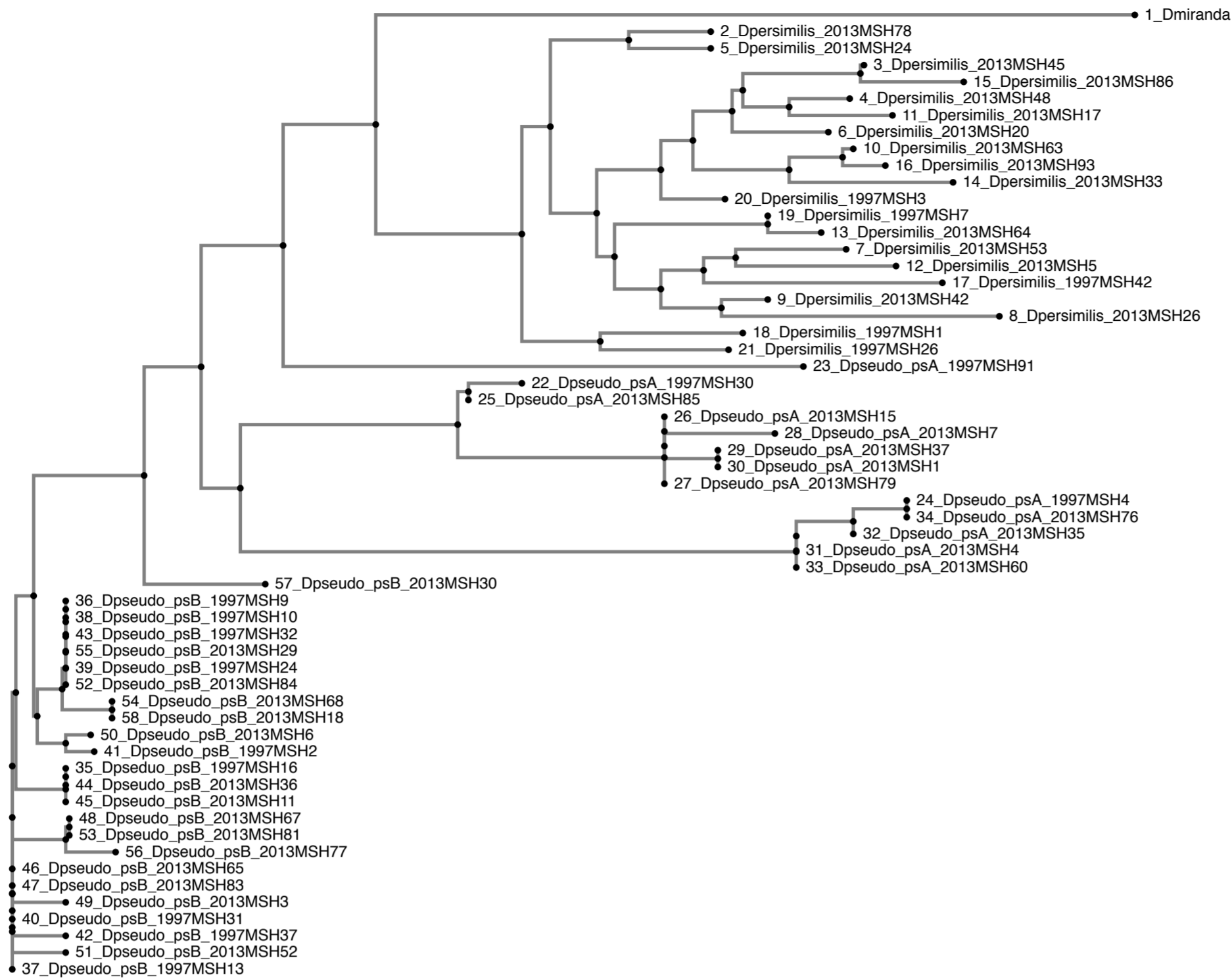

Supplement: S2 Fig — Independent clustering of psB and of psA with D. persimilis sequences can be seen in this neighbor joining phylogeny generated using phylo.io [23]. D. miranda is used as an outgroup. (PDF) [file pone.0165032.s002.pdf]
