# Supplementary material for: Mistaken Identity: Another Bias in the Use of Relative Genetic Divergence Measures for Detecting Interspecies Introgression
Source: PLoS One. 2016 Oct 19;11(10):e0165032. doi: 10.1371/journal.pone.0165032 (PMC5070774; doi:10.1371/journal.pone.0165032)
Supplement: S1 Table — (DOCX) [file pone.0165032.s003.docx]

**S1 Table. Presence of psA and psB haplotype structure in additional populations of *D. pseudoobscura*.**

|  | **psA** | **psB** | **Total** |
| --- | --- | --- | --- |
| Roslyn/Easton/Goldendale, WA | 10 | 8 | 18 |
| Cheney, WA | 7 | 8 | 15 |
| American Fork Canyon, UT | 2 | 6 | 8 |
| Flagstaff, AZ | 6 | 6 | 9 |
