# Supplementary material for: Mistaken Identity: Another Bias in the Use of Relative Genetic Divergence Measures for Detecting Interspecies Introgression
Source: PLoS One. 2016 Oct 19;11(10):e0165032. doi: 10.1371/journal.pone.0165032 (PMC5070774; doi:10.1371/journal.pone.0165032)
Supplement: S2 Table — (DOCX) [file pone.0165032.s004.docx]

**S2 Table. *In silico* expansion of individual psA haplotypes and comparison to *D. persimilis*, with indels excluded.**

| **Expanded Sequence**  **(psA-type)** | **Fixed Differences** | | **Tajima’s D*** | **D_a_**  **relative to *D. persimilis*** | **D_xy_**  **relative to *D. persimilis*** |
| --- | --- | --- | --- | --- | --- |
|  | **SNPs** | **indels** |  |  |  |
| 1997_MSH_pse30 | 7 | 0 | -0.24378 | 0.01262 | 0.01855 |
| 1997_MSH_pse91 | 10 | 3 | 1.5878 | 0.0179 | 0.02387 |
| 1997_MSH_pse4 | 17 | 0 | -0.0563 | 0.0251 | 0.03102 |
| 2013_MSH_pse1 | 9 | 0 | -0.57549 | 0.01541 | 0.02133 |
| 2013_MSH_pse4 | 15 | 0 | -0.28705 | 0.02265 | 0.02857 |
| 2013_MSH_pse7 | 9 | 0 | -0.48896 | 0.01541 | 0.02133 |
| 2013_MSH_pse15 | 8 | 0 | -0.80625 | 0.0142 | 0.02012 |
| 2013_MSH_pse35 | 16 | 0 | -0.1861 | 0.02387 | 0.0298 |
| 2013_MSH_pse37 | 9 | 0 | -0.57549 | 0.01544 | 0.02138 |
| 2013_MSH_pse60 | 15 | 0 | -0.28705 | 0.02265 | 0.02857 |
| 2013_MSH_pse76 | 17 | 0 | -0.0563 | 0.0251 | 0.03102 |
| 2013_MSH_pse79 | 8 | 1 | -0.80625 | 0.0142 | 0.02012 |
| 2013_MSH_pse85 | 8 | 0 | -0.14283 | 0.01383 | 0.01976 |
| Mean | 11.6923 | | -0.22492 | 0.01833 | 0.02426 |
| Median | 9 | | -0.28705 | 0.01544 | 0.02138 |
| Standard Deviation | 3.8384 | | 0.60211 | 0.00476 | 0.00476 |
| Maximum | 17 | | 1.5878 | 0.0251 | 0.03102 |
| Minimum | 7 | | -0.80625 | 0.01262 | 0.01855 |
| Range | 10 | | 2.39405 | 0.01248 | 0.01247 |

*Tajima’s D was calculated for all psA samples and expanded sequences, combined, to reiterate hypothetical expansion of psB haplogroup from a single haplotype.
