# Supplementary material for: Mistaken Identity: Another Bias in the Use of Relative Genetic Divergence Measures for Detecting Interspecies Introgression
Source: PLoS One. 2016 Oct 19;11(10):e0165032. doi: 10.1371/journal.pone.0165032 (PMC5070774; doi:10.1371/journal.pone.0165032)
Supplement: S3 Table — (DOCX) [file pone.0165032.s005.docx]

**S3 Table. *In silico* expansion of individual psA haplotypes and comparison to *D. persimilis*, with indels included.**

| **Expanded Sequence**  **(psA-type)** | **Fixed Differences** | **Tajima’s D*** | **D_a_**  **relative to *D. persimilis*** | **D_xy_**  **relative to *D. persimilis*** |
| --- | --- | --- | --- | --- |
| 1997_MSH_pse30 | 7 | -0.63866 | 0.01267 | 0.01928 |
| 1997_MSH_pse91 | 14 | -0.00019 | 0.02307 | 0.02968 |
| 1997_MSH_pse4 | 17 | 0.05897 | 0.02557 | 0.03219 |
| 2013_MSH_pse1 | 9 | -0.37605 | 0.01628 | 0.02289 |
| 2013_MSH_pse4 | 15 | -0.05012 | 0.02313 | 0.02974 |
| 2013_MSH_pse7 | 9 | -0.46097 | 0.01544 | 0.02205 |
| 2013_MSH_pse15 | 8 | -0.52991 | 0.01423 | 0.02085 |
| 2013_MSH_pse35 | 16 | 0.0052 | 0.02435 | 0.03096 |
| 2013_MSH_pse37 | 9 | -0.37605 | 0.01632 | 0.02295 |
| 2013_MSH_pse60 | 15 | 0.02749 | 0.02398 | 0.0306 |
| 2013_MSH_pse76 | 17 | 0.05897 | 0.02557 | 0.03219 |
| 2013_MSH_pse79 | 9 | -0.46097 | 0.01544 | 0.02205 |
| 2013_MSH_pse85 | 8 | -0.48061 | 0.01471 | 0.02133 |
| Mean | 11.76923077 | -0.247915385 | 0.019289231 | 0.025904615 |
| Median | 9 | -0.37605 | 0.01632 | 0.02295 |
| Maximum | 7 | 0.05897 | 0.02557 | 0.03219 |
| Minimum | 17 | -0.63866 | 0.01267 | 0.01928 |
| Range | 10 | 0.69763 | 0.0129 | 0.01291 |

*Tajima’s D was calculated for all psA samples and expanded sequences, combined, to reiterate hypothetical expansion of psB haplogroup from a single haplotype.
